# From Mother to Child: Epigenetic Signatures of Hyperglycemia and Obesity during Pregnancy

**DOI:** 10.3390/nu16203502

**Published:** 2024-10-16

**Authors:** Marica Franzago, Paola Borrelli, Marta Di Nicola, Pierluigi Cavallo, Ebe D’Adamo, Luciano Di Tizio, Diego Gazzolo, Liborio Stuppia, Ester Vitacolonna

**Affiliations:** 1 Department of Medicine and Aging, School of Medicine, and Health Sciences, “G. D’Annunzio” University, Via dei Vestini, Chieti-Pescara, 66100 Chieti, Italy; marica.franzago@unich.it (M.F.); diego.gazzolo@unich.it (D.G.); 2 Center for Advanced Studies and Technology (CAST), “G. D’Annunzio” University, Chieti-Pescara, 66100 Chieti, Italy; stuppia@unich.it; 3 Laboratory of Biostatistics, Department of Medical, Oral and Biotechnological Sciences, “G. D’Annunzio” University, Chieti-Pescara, 66100 Chieti, Italy; paola.borrelli@unich.it (P.B.); marta.dinicola@unich.it (M.D.N.); 4 Neonatal Intensive Care Unit, “G. D’Annunzio” University, 66100 Chieti, Italy; ebe.dadamo@yahoo.com; 5 Department of Obstetrics and Gynaecology, SS. Annunziata Hospital, “G. D’Annunzio” University, 66100 Chieti, Italy; lucianoditizio@virgilio.it; 6 Department of Psychological, Health and Territorial Sciences, School of Medicine and Health Sciences, “G. D’Annunzio” University, Chieti-Pescara, 66100 Chieti, Italy

**Keywords:** gestational diabetes, epigenetic modifications, obesity, DNA methylation, *MC4R*, *LPL*, fetal programming, gene expression

## Abstract

Background: In utero exposure to maternal hyperglycemia and obesity can trigger detrimental effects in the newborn through epigenetic programming. We aimed to assess the DNA methylation levels in the promoters of *MC4R* and *LPL* genes from maternal blood, placenta, and buccal swab samples collected in children born to mothers with and without obesity and Gestational Diabetes Mellitus (GDM). Methods: A total of 101 Caucasian mother–infant pairs were included in this study. Sociodemographic characteristics, clinical parameters, physical activity, and adherence to the Mediterranean diet were evaluated in the third trimester of pregnancy. Clinical parameters of the newborns were recorded at birth. Results: A negative relationship between *MC4R* DNA methylation on the fetal side of the GDM placenta and birth weight (r = −0.630, *p* = 0.011) of newborns was found. *MC4R* DNA methylation level was lower in newborns of GDM women (CpG1: 2.8% ± 3.0%, CpG2: 3.8% ± 3.3%) as compared to those of mothers without GDM (CpG1: 6.9% ± 6.2%, CpG2: 6.8% ± 5.6%; *p* < 0.001 and *p* = 0.0033, respectively), and it was negatively correlated with weight (r = −0.229; *p* = 0.035), head circumference (r = −0.236; *p* = 0.030), and length (r = −0.240; *p* = 0.027) at birth. *LPL* DNA methylation was higher on the fetal side of the placenta in obese patients as compared to normal-weight patients (66.0% ± 14.4% vs. 55.7% ± 15.2%, *p* = 0.037), and it was associated with maternal total cholesterol (r = 0.770, *p* = 0.015) and LDL-c (r = 0.783, *p* = 0.012). Conclusions: These results support the role of maternal *MC4R* and *LPL* methylation in fetal programming and in the future metabolic health of children.

## 1. Introduction

Chronic non-communicable diseases (NCDs), also known as chronic diseases, are influenced by a combination of genetic, physiological, environmental, and behavioral factors throughout life and represent the leading cause of morbidity and mortality worldwide [1].

The risk of developing NCDs is also in early life, and well-established evidence has shown that pregnancy and infancy are the most critical stages [2], hence the concept of ‘fetal programming’. Early environmental exposure to maternal conditions may be a key reason for the offspring’s impaired health in adulthood [3]. Epigenetics, which refers to the changes in gene expression without the modification of the nucleotide sequence, provides a relationship between genes and the environment, and epigenetic changes can be inherited by the future generation with persisting variations in gene regulation and in a range of metabolic as well as physiological pathways [4]. To date, several studies suggested that the risk of NCDs may be induced by epigenetic alterations since genetics contributes only a small proportion of the effects [5]. DNA methylation of specific sequences in promoter regions is one of the most stable and best-understood epigenetic modifications. Several studies show that epigenetic modifications related to maternal metabolic and nutritional conditions could contribute to explaining the detrimental health effects of the newborn associated with fetal programming, including increased risk of obesity and type 2 diabetes mellitus in childhood and later life [6]. Among the environmental factors, maternal lifestyle habits play a crucial role in the programming of metabolic diseases early in life. The global prevalence of obesity in pregnancy and Gestational Diabetes Mellitus (GDM) is rapidly increasing, and these common conditions can potentially induce short- and long-term consequences in both mother and baby [7]. GDM is defined as any degree of glucose intolerance that was first recognized during pregnancy but that was not clearly overt diabetes [8] and is often associated with obesity. In fact, obesity is a risk factor for GDM, and maternal body mass index (BMI) is the most important modifiable risk factor for GDM onset, with a 6.79-fold increased risk in obese and 2.29-fold increased risk in overweight women [9]. Some modifiable risk factors that have been identified for GDM include an unhealthy dietary factor, cigarette smoking, and physical inactivity [10]. Moreover, some studies have shown how maternal obesity and GDM constitute serious risk factors for the development of NCDs in the offspring. The rise in the global prevalence of maternal obesity and GDM has emphasized the need for a greater understanding of the pathophysiological mechanisms that underlie these conditions. In addition, there is growing interest in understanding the effects of maternal conditions on offspring metabolic health via epigenetic mechanisms. Some studies provided significant findings on alterations of DNA methylation patterns in hyperglycemic and obesogenic maternal–fetal conditions [4,11].

The central melanocortin pathways play a critical role in energy homeostasis and in regulating body weight. Melanocortin 4 receptor (*MC4R*) activity is crucial for the regulation of metabolism and food intake, and the peptide ligands for the MC4R are associated with feeding, energy expenditure, and complex behaviors that orchestrate energy intake and expenditure [12]. Previous studies have shown that the hypothalamic melanocortin system plays a pivotal role in the developmental programming induced by maternal dietary manipulation models [13,14]. Thus, impaired perinatal development may induce metabolic consequences sensitizing the offspring to long-lasting energy balance dysfunction. Given that the *MC4R* gene promoter region is regulated by epigenetic modifications following nutritional status [15], it would also be important to examine the epigenetic mechanisms in offspring.

Moreover, lipoprotein lipase (LPL) is involved in metabolic homeostasis through triglyceride hydrolysis and plays an important role in lipid metabolism and transport. The fetus is unable to synthetize enough free fatty acids (FFA) as a result of hydrolysis and thus depends on transplacental transfer. Obesity combined with pregnancy can trigger an increase in maternal circulating lipids with advancing gestation, resulting in excess lipid transfer to the developing fetus via placental LPL activity [16]. Therefore, the increase in fetal lipid exposure can induce the development of metabolic disease in childhood, affecting the liver, skeletal muscle, adipose tissue, and pancreas [16].

Interestingly, in the study by Gagné-Ouellet [17], a significant decrease related to placental DNA methylation level in the CpG1 and CpG2 of the *LPL* gene in GDM women was demonstrated. 

According to the concept of fetal metabolic programming through epigenetic changes, the study also demonstrated that DNA methylation at the *LPL* gene in fetal placental was associated with the anthropometric parameters in 5-year-old children.

Thus, given the prominent role of genes *MC4R* and *LPL* in energy and metabolic homeostasis and since an adverse intrauterine environment (such as hyperglycemia and maternal obesity during pregnancy) can affect the DNA methylation pattern both in mothers and their offspring, the effects of DNA methylation in the promoters of these two candidate genes from some tissue, including placenta, maternal blood, and buccal swab samples collected in children born to women with and without obesity and GDM, were explored.

## 2. Materials and Methods

### 2.1. Study Design and Participants

For this cross-sectional study, 101 newborns who were born to Caucasian women were recruited. Only singleton, viable infants are included in this study. Clinical parameters and buccal swab samples of newborns were collected at the Department of Neonatology and Neonatal Intensive Care Unit of University “Gabriele d’Annunzio” of Chieti. This project, conducted in accordance with the Declaration of Helsinki, was approved by the Ethics Committee of the Province di Chieti and Pescara (date of approval: 8 April 2021). All mothers gave their written consent before their inclusion in this study.

### 2.2. Inclusion and Exclusion Criteria

Women over 18 years of age who delivered singletons were included in this study.

According to the International Association of Diabetes and Pregnancy Study Groups (IADPSG) criteria, Gestational Diabetes Mellitus (GDM) diagnosis was proved both at the 16–18th and/or at the 24–28th weeks of gestation [18]. Mothers diagnosed with GDM and/or obesity were advised Medical Nutritional Therapy (MNT) and exercise.

Women with the following diagnoses were excluded from this study: pre-gestational type 1 or 2 diabetes, overt diabetes, hypertension, a history of drug or alcohol abuse, or other chronic diseases such as kidney and liver diseases.

### 2.3. Maternal Anthropometric, Clinical Data, and Sampling

Data on sociodemographic characteristics, as well as clinical (fasting plasma glucose, lipid profile, and blood pressure at the third trimester of pregnancy) and anthropometric parameters (pre-pregnancy BMI and, at the end of the pregnancy, gestational weight gain), were collected. Blood samples from each woman were collected and stored at −20 °C until DNA extraction (for genetic and DNA methylation analyses).

#### Maternal Lifestyle Questionnaires

Mediterranean diet adherence, smoking habits, and physical activity (PA) of the women were assessed in the third trimester of pregnancy. International Physical Activity Questionnaire (IPAQ-short version) was used to measure PA [19], recording the activity of three intensity levels (low, moderate, and high PA). The 14-point Mediterranean Diet Adherence Screener (MEDAS) questionnaire from the PREDIMED study comprises 14 items regarding the main groups of food commonly consumed in the Mediterranean area, recording the frequency of consumption of the food items in servings per day or week [20]. The final 14-MEDAS score, which ranges between 0 and 14, applies the following criteria: (i) no adherence, ≤5; (ii) moderate to fair adherence, 6–9; (iii) maximum adherence to the Med-Diet, ≥10 [17,21].

### 2.4. Perinatal Outcomes and Newborn’s Sampling

The following main perinatal and neonatal outcomes were recorded at birth: gestational age, delivery mode (vaginal delivery, cesarean section), gender, Apgar scores at 1st and 5th min, birth weight, and length and head circumference. 

Neonatal blood glucose concentrations were measured on capillary blood samples taken by heel-prick lance and analyzed on a blood gas analyzer using the glucose oxidase method. Capillary glucose measurement was performed within the first hours of birth. Hypoglycemia was defined according to current criteria [22].

Buccal swab samples were collected in all newborns at birth and stored at −20 °C until DNA extraction (for genetic and DNA methylation analyses).

The newborn’s buccal swab was chosen since it has technical advantages over blood, particularly for its non-invasive sampling method and molecular measures in buccal swabs, which might reflect those in blood [23].

### 2.5. Molecular Studies

#### 2.5.1. Genotyping

Genomic DNA was isolated from peripheral blood lymphocytes in women and from buccal swabs in newborns using the MagPurix 12s Automated Nucleic Acid Purification System (Zinexts Life Science Corp., New Taipei City, Taiwan).

Therefore, genetic variant rs326 (*A* > *G*) in *LPL* gene, previously identified as associated with lipid metabolism, was selected [24,25,26].

The rs326 was genotyped in all pregnant women and their offspring. In detail, after extraction, nucleic acid concentrations were measured using the Qubit assay kit on a Qubit 4 Fluorometer (Invitrogen, Thermo Fisher, Waltham, MA, USA). The DNA samples were amplified by polymerase chain reaction (PCR) in 30 μL reaction volume containing 30 ng of genomic DNA using AmpliTaq Gold DNA Polymerase in a SimpliAmp™ thermal cycler (Applied Biosystems™, Waltham, MA, USA). PCR conditions involved an initial denaturation at 95 °C for 10 min, followed by 35 cycles of denaturation at 95 °C for 30 s, annealing at 59 °C for 30 s, and extension at 72 °C for 30 s, concluding with a final extension at 72 °C for 10 min. 

The amplified products (499 base pairs) were then subjected to direct sequencing using BigDye Term v3.1 Cycle Sequencing Kit (Life Technologies, Monza, Italy) and subsequently analyzed via automatic sequencing on SeqStudio™ Genetic Analyzer (Applied Biosystems™, Waltham, MA, USA). Specific PCR primers (*LPL* F: 5′-TACACTAGCAATGTCTAGCTGA-3′ and *LPL* R: 5′-TCAGCTTTAGCCCAGAATGC-3′) were designed based on the reference gene sequence.

#### 2.5.2. Placenta Tissue Sampling (DNA and Total RNA Extraction)

Forty placentas for DNA and RNA extraction from pregnancies complicated by GDM or obesity, as well as from healthy pregnancies, were selected. Placenta biopsies were obtained by trained medical personnel within a few minutes after delivery and placenta expulsion. After removal of the decidua, four biopsies, each with a volume of 0.5 cm^3^ (two for DNA and two for RNA analysis), were collected from both the fetal and maternal sides. The samples were washed with 1X PBS to eliminate cord/maternal blood and dissected to remove conjunctive tissues.

The tissue samples were preserved in RNAlater Stabilization Solution (Thermo Fischer Scientific, Waltham, MA, USA) and stored at −80 °C until further analysis. DNA was extracted from the fetal and maternal sides of the placenta using the MagPurix 12s Automated Nucleic Acid Purification System (Zinexts Life Science Corp., New Taipei City, Taiwan) in accordance with the manufacturer’s instructions, as previously reported in the literature [11,27]. In addition, 10 mg of placental tissue from the fetal and maternal sides was sonicated using a custom tool to mince the tissue and facilitate the release of RNA into the solution. For RNA purification, the Total RNA Purification Kit (17270) (Norgen Biotek Corp, Thorold, Canada) was employed. The protocol begins with the preparation of the lysate from the tissue using Buffer RL, which is provided with the kit. After lysis, the RNA was bound to a column containing a resin that exploits ionic concentrations to selectively bind the RNA while proteins and other cellular contaminants were removed. The RNA purification process proceeds through a series of column washes, followed by the final elution of the RNA in an elution buffer. The entire extraction process was conducted in an RNase-free environment, adopting stringent measures to prevent contamination and degradation of the RNA.

DNA and RNA concentrations were quantified by measuring ultraviolet (UV) absorption using the NanoPhotometer NP80 instrument (Implen, Inc., Westlake Village, CA, USA). Additionally, the purity of both DNA and RNA was assessed by evaluating the absorbance ratio at 260 nm to 280 nm.

#### 2.5.3. Epigenetic Analysis (DNA Methylation Analysis in Mothers, Newborns, and Placenta)

**Figure 1:**
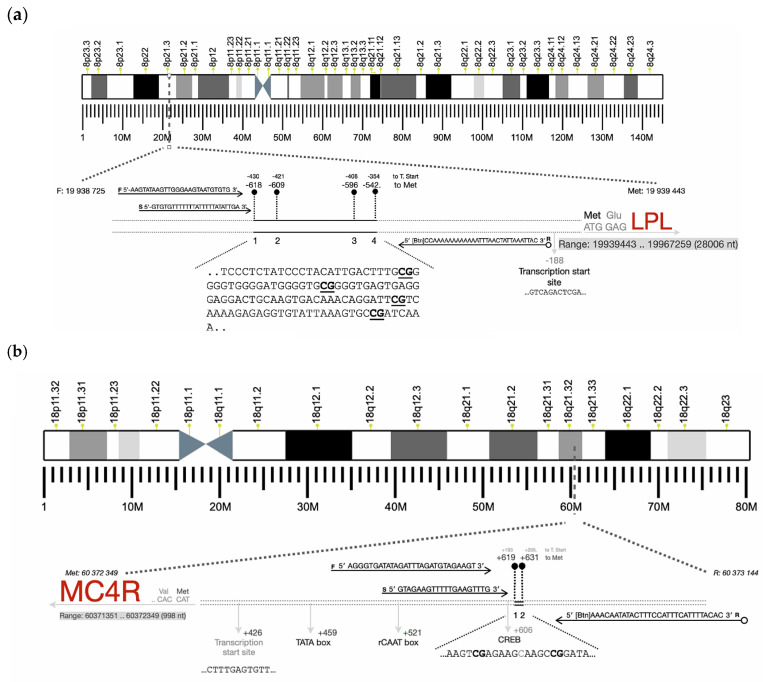
Schematic representation of the genes analyzed and localization of the CpGs ((**a**): *LPL* gene; (**b**): *MC4R* gene).

DNA methylation in *MC4R* and *LPL* promoter regions in placenta, peripheral blood, and buccal cells were analyzed. Specific CpG sites within the promoter regions (Figure 1a,b) were selected and assessed using pyrosequencing (Qiagen, Pyromark Q48 Autoprep). Pyrosequencing technology, based on the sequencing-by-synthesis principle, enables accurate quantification of DNA sequences by measuring nucleotide incorporation. Specifically, during DNA synthesis, the addition of one of the four nucleotides results in the release of pyrophosphate, which triggers enzymatic reactions producing light. The intensity of the emitted light is directly proportional to the amount of incorporated nucleotides [28].

The pyrosequencing assay design was performed using Pyrosequencing Assay Design Software v2.0 (Qiagen, Hilden, Germany), which enables efficient design of PCR amplification and sequencing primers. After providing the target sequence, the software assisted in the automated selection of target CpGs, generating the rate the success chance of each generated assay, optimizing coverage and assay specificity for each analyzed locus, minimizing potential interference (including primer length and positioning, GC content, and potential stem-loops). PCR amplification and sequencing primers are presented in Table 1.

Genomic DNA was modified by sodium bisulfite using *the EpiTect Plus DNA Bisulfite Kit* (Qiagen, Hilden, Germany). Bisulfite-treated DNAs were amplified by PCR using *the Pyromark PCR kit (200) (Qiagen)* according to the following program: denaturation at 95 °C for 15 min, followed by 45 cycles at 94 °C, 60 °C (for *MC4R*) −59 °C (for *LPL*), and 72 °C, each for 30 s, and a final extension cycle at 72 °C for 10 min. After amplification, the specificity of PCR products was then verified by electrophoresis. Finally, the PCR products were sequenced by the PyroMark Q48 Autoprep (Qiagen) using PyroMark Q48 Advanced CpG Reagents reagents (Qiagen, Hilden, Germany), according to manufacturer’s recommendations.

The methylation level of each CpG site was computed using PyroMark Q48 Autoprep 2.4.2. software (Qiagen, Hilden, Germany), which calculates the ratio of the peak height of methylated cytosine to unmethylated thymine, providing an accurate percentage of methylation for each CpG site analyzed. 

#### 2.5.4. Gene Expression Analysis in Placenta

To evaluate the effects of DNA methylation on the expression of the *LPL* and *MC4R* genes in placental tissue, mRNA levels were measured.

The gene expression analysis of *MC4R* and *LPL* was conducted using the TaqMan assay. This technique is based on the use of specific TaqMan probes. The procedure consists of two stages: the RNA reverse transcription to cDNA and subsequent amplification. During the extension phase, if the TaqMan probe is annealed to its specific target, it cleaves the probe between the fluorophore and the quencher along the new DNA synthesis. This cleavage causes the positive fluorophore to move away from the quencher (negative fluorophore), allowing the positive fluorophore to emit light. 

Reverse transcription was conducted using the High-Capacity cDNA Reverse Transcription Kit (Applied Biosystems, Waltham, MA, USA) following the manufacturer’s protocol. Each 10 µL reaction contained 2 µL of 10× RT Buffer, 0.8 µL of 25× dNTP Mix, 2 µL of 10× RT Random Primers, 1 µL of MultiScribe™ Reverse Transcriptase, and 3.2 µL of nuclease-free water. Subsequently, 2 µg of purified RNA dissolved in 10 µL was added to each reaction mixture. The synthesized cDNA was then amplified using the TaqMan Fast Advanced Master Mix (Applied Biosystems, Waltham, MA, USA) according to the manufacturer’s instructions. The 18 µL amplification mixture comprised 10 µL of 2× TaqMan Fast Advanced Master Mix, 1 µL of TaqMan Assay, and 7 µL of nuclease-free water. To each amplification reaction, 2 µL of the previously reverse-transcribed cDNA was added. The prepared mixtures were dispensed into a PCR plate, which was subsequently sealed with MicroAmp Optical Adhesive Film (Applied Biosystems, Waltham, MA, USA) and centrifuged using an Eppendorf 5810 centrifuge to ensure proper reagent distribution.

The plate was loaded into QuantStudio 5 Real-Time PCR System (Applied Biosystem, Foster City, CA, USA), previously configured with the following thermal cycle conditions: Polymerase activation 20 s (denature at 95 °C for 1 s, anneal/extend at 60 °C for 20 s) for 40 cycles. At the end of each cycle, the instrument detected the increase in fluorescence line the amplification plot. The samples were loaded in duplicate for each run. In agreement with the literature, succinate dehydrogenase complex, subunit A (*SDHA),* served as the housekeeping gene to normalize expression levels across the analyzed sample population during the analytical phase. In fact, the literature demonstrated that using *SDHA,* which exhibits greater expression stability compared to the housekeeping genes typically employed in placental studies, enhances both the sensitivity and specificity of gene expression profile analyses [29]. Results were analyzed using Design & Analysis Software 2 (Thermo Fisher) and calculated according to the 2^−ΔΔCt^ method.

### 2.6. Statistical Analysis

Descriptive analysis used mean and standard deviation (SD) or median and interquartile range (IQR) for the quantitative variables and percentage values for the qualitative ones. Statistical units were mothers and newborns, who were independently analyzed. The Shapiro–Wilk Test assessed normality distribution for quantitative variables. The association between categorical data was investigated by Pearson χ2 or Fisher’s exact test and the Student’s *t*-test for independent data or Analysis of Variance (ANOVA) for more than two groups for continuous data with subsequent post hoc Bonferroni test or non-parametric analog tests (Wilcoxon rank sum test or Kruskal–Wallis test). In addition, Benjamini–Hochberg’s false discovery rate correction for multiple comparison tests was applied. The relationships among quantitative variables were tested using Pearson’s correlation coefficient (r). Hardy–Weinberg equilibrium was calculated. A statistical significance was set at the level of ≤0.05 unless adjustment for multiple comparisons was needed. All analyses were performed using Stata software v18.0 MP (StataCorp, College Station, TX, USA).

## 3. Results

### 3.1. Characteristics of the Study Population

Data were collected for 101 mother–infant pairs. The average age of the 101 women was 35.0 (±5.0) years. Additionally, 54.46% of women had a normal pre-pregnancy BMI (BMI ≥ 18.5 and <25 kg/m^2^). About 93.0% of mothers had an education level of college or higher. Additionally, 42.9% of women reported being smokers or ex-smokers during pregnancy.

Of the 101 recruited mothers, 45.54% were obese or overweight (OB), and 83.2% had a GDM diagnosis [2 h post-75 g oral glucose tolerance test (OGTT) glucose] according to IADPSG criteria [18]. Moreover, 55.3% of GDM mothers were treated with a diet only.

Infants were born at an average gestational age of 38.6 (±1.2) weeks, and 43% of them were girls. The majority of the infants (63%) were born by vaginal delivery. The mean neonatal birth weight was 3215.7 (±435.3) gr, and the neonatal birth length was 49.7 (±1.9) cm.

### 3.2. Anthropometric and Clinical Data of GDM versus Normoglycemic (NGT) Women

Eighty-four consecutive women with GDM (mean age 35.3 ± 4.9 years) and seventeen nondiabetic pregnant consecutive subjects (mean age 33.7 ± 5.3 years) were assessed in this cohort. Mean glucose values according to GDM status are presented in Table 2. Among GDM women, 54.8% of MNT was not enough to ensure adequate glycemic control; thus, insulin treatment became necessary.

The no-GDM group, when compared to the GDM, showed a significantly higher increase in weight at delivery (92.45 ± 5.49 vs. 80.49 ± 1.93, *p* = 0.019). GDM women showed higher serum concentrations of TC (254.3 ± 46.3 vs. 225.6 ± 30.3) mg/dL, *p* = 0.049) during the third trimester than the NGT group. Regarding lifestyle habits, the median PREDIMED score was 8.0 (7–9 IQR) and 9.0 (8–10 IQR) for NGT vs. GDM women, respectively (*p* = 0.037).

### 3.3. Anthropometric and Clinical Data of OB versus Normal Weight (NW) Pregnant Women

A total of 46 consecutive OB (mean age 34.4 ± 5.6 years) and 55 consecutive normal-weight pregnant women (mean age 34.6 ± 4.3 years) were assessed in this cohort. Regarding lifestyle habits, the median PREDIMED score was 9.5 (9–10 IQR) and 9.0 (8–10 IQR) for NW and OB women, respectively (*p* = 0.004). In fact, OB showed lower adherence to the Med-Diet when compared to NW (*p* = 0.013) (Table 2).

OB patients showed higher BMI both in the pre-pregnancy stage (33.3 ± 7.3 vs. 21.8 ± 1.9 Kg/m^2^, *p* < 0.001) and at the end of pregnancy (36.1 ± 6.9 vs. 26.2 ± 2.4 Kg/m^2^, *p* < 0.001) than the NW group. In addition, the OB group, when compared to the NW group, showed a significantly lower increase in average weight gain at delivery vs. the weight recorded before pregnancy (7.5 ± 6.6 vs. 11.7 ± 5.9), *p* < 0.001). They also showed higher systolic blood pressure (121.2 ± 15.1 vs. 112.2 ± 11.6 mmHg, *p* = 0.005) and diastolic blood pressure (73.3 ± 8.6 vs. 67.7 ± 7.9 mmHg, *p* = 0.005) when compared with controls. Moreover, OB women showed lower serum concentrations of HDL-C (59.2 ± 11.7 vs. 68.1 ± 12.7 mg/dL, *p* = 0.001) during the 3rd trimester than the control group. Finally, OB women showed higher blood glucose values at fasting during the 75 g oral glucose tolerance test (OGTT) compared to NW women (93.0 ± 7.8 vs. 86.6 ± 9.2 mg/dL, *p* < 0.001).

## 4. Neonatal Characteristics

### 4.1. Neonatal Outcomes Relative to GDM and NGT Pregnant Women

The main neonatal anthropometric characteristics relative to NGT and GDM women are summarized in Table 3. Mean gestational age at delivery was significantly lower in the GDM group (38.5 ± 1.2 vs. 39.1 ± 1.4, *p* = 0.047). In addition, the Apgar score at 1 min was 8.8 ± 0.7 and 7.9 ± 1.7, *p* < 0.001, and the Apgar score at 5 min was 9.8 ± 0.7 and 9.2 ± 0.9, *p* < 0.001 for GDM and NGT women, respectively. With regard to blood glucose values, hypoglycemia occurred more frequently in newborns of women with GDM than NGT (44.9% vs. 18.8%, *p* = 0.050).

### 4.2. Neonatal Outcomes Relative to OB and NW Pregnant Women

The main neonatal anthropometric characteristics relative to NW and OB women are summarized in Table 3. Mean gestational age at delivery was significantly lower in the OB group than NW pregnant women (38.2 ± 1.0 vs. 38.9 ± 1.3, *p* = 0.014). Newborns of OB women showed lower Apgar scores at 1 min (8.4 ± 1.3 vs. 8.8 ± 0.5), *p* = 0.028) than newborns of the control group.

## 5. Genetic Analysis in Mothers and Newborns

**Figure 2:**
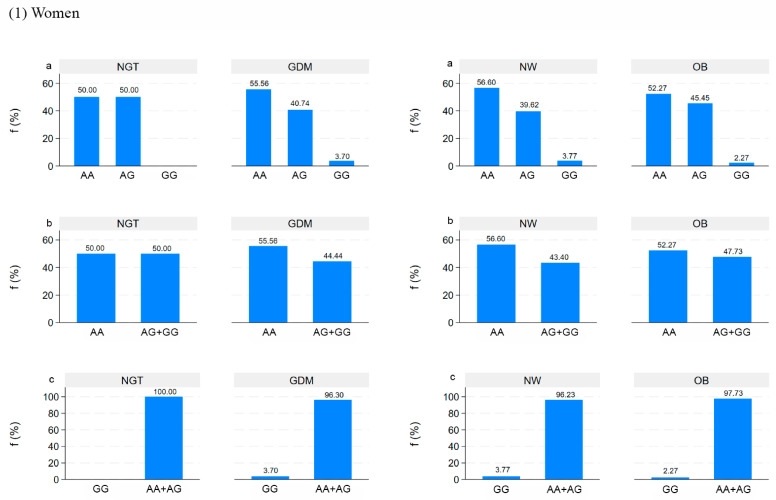

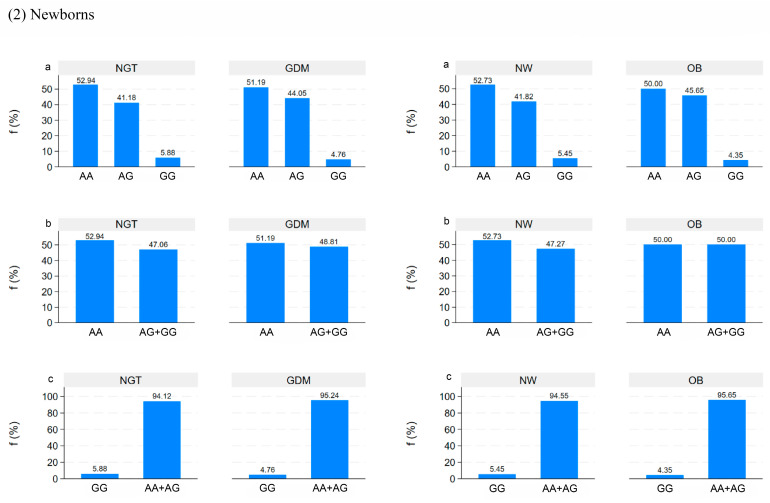
The genotypes distribution of the *LPL* rs326 based on additive (**a**), dominant (**b**), and recessive (**c**) inheritance genetic models in women and newborns by NGT/GDM and NW/OB groups.

The genotype distribution of rs326 (*A* > *G*) in the *LPL* gene in all pregnant women and their offspring is reported in Figure 2a,b based on additive, dominant, and recessive inheritance genetic models. The genotype frequencies were within the Hardy–Weinberg equilibrium (*p* > 0.05).

No difference in the frequency of rs326 between NGT-GDM and NW-OB was shown.

In addition, no relations between *LPL* rs326 in the inheritance genetic models and clinical data of mothers (pre-pregnancy weight, weight at the end of pregnancy, pre-pregnancy BMI, BMI at the end of pregnancy, lipid profile) and newborns (birth weight and length) were found.

### 5.1. Correlation between DNA Methylation and Clinical Data in Mothers and Newborns

*MC4R* DNA methylation levels at CpG1 and CpG2 were lower in newborns of GDM women as compared to newborns of mothers without GDM (2.8% ± 3.0% vs. 6.9% ± 6.2%), *p* < 0.001 and 3.8% ± 3.3% vs. 6.8% ± 5.6%), *p* = 0.003, respectively) (Table 4).

Maternal *MC4R* DNA methylation at CpG1 and CpG2 was negatively related to BMI at the end of pregnancy in GDM women (r = −0.220, *p* = 0.047 and r = −0.241, *p* = 0.029, respectively). 

In addition, *MC4R* methylation levels at CpG1 in newborns of GDM women were negatively correlated with weight (r = −0.229, *p* = 0.035), head circumference (r = −0.236, *p* = 0.030), and length (r = −0.240, *p* = 0.027) at birth.

On the other hand, no difference between DNA methylation levels at *LPL* in mothers and newborns was observed.

### 5.2. Correlation between DNA Methylation in Placenta and Clinical Data in Mothers and Newborns

Forty placentas from GDM or obesity-complicated pregnancies and healthy pregnancies were analyzed.

Placental *LPL* DNA methylation levels at CpG4 were higher on the fetal side of obese placentas as compared to normal weight (66.0% ± 14.4% vs. 55.7% ± 15.2%, *p* = 0.037) (Supplementary Table S1).

Related to obese patients, the mean placental *LPL* methylation on the fetal side was associated with TC (r = 0.770, *p* = 0.015) and LDL (r = 0.783, *p* = 0.012) in the third trimester of pregnancy, respectively.

On the other hand, no difference between DNA methylation levels at *LPL* and *MC4R* on the maternal and fetal sides of the placentas in women with and without GDM was observed. Nevertheless, DNA methylation levels at *LPL*-CpG1 on the maternal side in GDM women were negatively correlated with the birth length of newborns (r = −0.642, *p* = 0.009). In addition, the mean of DNA methylation levels at *LPL* on the fetal side was also correlated with pre-pregnancy BMI (r = 0.812, *p* < 0.001) and BMI at the end of pregnancy (r = 0.884, *p* < 0.001) in GDM. Moreover, a negative relation between fetal mean methylation levels at *MC4R* and birth weight (r = −0.630, *p* = 0.011) of newborns was found.

### 5.3. Correlation between Placental LPL and MC4R DNA Methylation and mRNA Expression Levels in Placenta

mRNA levels were calculated according to the 2^−ΔΔCt^ method. *LPL* DNA methylation levels at CpG4 on the fetal side of the placenta were negatively correlated with placental *LPL* gene expression (r = −0.35 *p* = 0.029). In addition, *MC4R* DNA methylation levels on the fetal side of the placenta in GDM women were negatively correlated with placental *MC4R* gene expression (r = −0.47 *p* = 0.036). Moreover, *LPL* DNA methylation levels at CpG4 on the fetal side of the placenta in GDM women were negatively correlated with placental *LPL* gene expression (r = −0.47 *p* = 0.030).

## 6. Discussion

Exposure to adverse maternal conditions in pregnancy has been shown to trigger short- and long-term complications for the newborn via epigenetic mechanisms [30]. In particular, epigenetic modifications can contribute to the intergenerational inheritance of obesity, diabetes, and cardiovascular diseases. In this view, the aim of this study was to evaluate the relationship between exposure to an altered intrauterine environment and fetal metabolic programming, focusing on genetic and epigenetic effects from some tissue, including placenta, maternal blood, and buccal swab samples collected in children born to women with and without obesity and GDM. At first, our results showed a negative relation between the mean methylation levels at *MC4R* on the fetal side of the GDM placenta and birth weight (r = −0.630, *p* = 0.011) of newborns. Moreover, *MC4R* DNA methylation levels at CpG1 and CpG2 were lower in newborns of women with GDM compared to those without GDM (*p* < 0.001 and *p* = 0.003, respectively). 

These results evoke our previous study [27], which has shown, for the first time, that DNA methylation in the promoter of the *MC4R* gene on the fetal side of the placenta in GDM-affected women was lower than in non-GDM-affected recruits.

In this study, *MC4R* DNA methylation on the fetal side in GDM women was also negatively correlated with placental *MC4R* gene expression (r = −0.47, *p* = 0.036), which highlights the regulation of transcriptional activity. In this light, our previous result appears in agreement also with these new data showing that *MC4R* methylation levels in newborns of GDM women were negatively correlated with weight (r = −0.229, *p* = 0.035) head circumference (r = −0.236, *p* = 0.030) and length (r = −0.240, *p* = 0.027) at birth.

MC4R is a seven-transmembrane, G-protein-coupled receptor that regulates the feelings of fullness and hunger. In particular, MC4R is a key element in the hypothalamic control of food intake by participating in appetite, energy control, and glucose metabolism [31,32,33,34,35]. MC4R defects, which can disrupt the melanocortin pathway, lead to a clinical phenotype characterized by an increase in appetite, lack of satiety, and early-onset obesity [36]. Kwon et al. [37] showed that the *MC4R* methylation at birth was associated with metabolic profiles in childhood, suggesting that DNA methylation level may be predictive of the risk of developing metabolic syndrome. To date, further studies with long-term follow-up are needed to evaluate whether alterations in *MC4R* DNA methylation at birth also contribute to the development of consequences in adults.

Lipoprotein lipid physiology in pregnancy has significant implications for the mother, the developing fetus, the newborn, and their future health [38].

LPL contributes to the transfer of free fatty acids from maternal lipoproteins to the fetus. 

In our study, placental *LPL* DNA methylation levels at CpG4 were higher on the fetal side of obese placentas as compared to normal weight (*p* = 0.037) were observed.

A negative correlation between *LPL* DNA methylation levels at CpG4 and mRNA levels on the fetal side of the placenta (r = −0.35 *p* = 0.029) was found. Moreover, the mean placental *LPL* methylation on the fetal side in obese women was associated with TC (r = 0.770, *p* = 0.015) and LDL (r = 0.783, *p* = 0.012) in the third trimester of pregnancy, respectively. Furthermore, related to GDM women, the mean of DNA methylation levels at *LPL* on the fetal side were also correlated with pre-pregnancy BMI (r = 0.812, *p* < 0.001) and BMI at the end of pregnancy (r = 0.884, *p* < 0.001). 

Taking into account that LPL is a key player in lipoprotein metabolism, our findings reported the correlation between DNA methylation at *LPL* and maternal lipid profile in the third trimester.

Nevertheless, it is possible to suppose that *LPL* DNA methylation might be involved in the regulation of the placental lipid transfer to the fetus, but the exact effect of the maternal metabolism in regulating the placental epigenetic mechanisms needs to be clarified.

In fact, regarding our findings related to the correlation between DNA methylation in the placenta and clinical data in mothers and newborns, it should be noted that placental responses to maternal perturbations are complex and remain poorly understood. In particular, perturbations mediated by the placenta action can influence fetal development and can predispose to diseases later in life. As a matter of fact, the placenta, which represents the primary barrier to maternal–fetal exchange, plays a critical role in receiving and mediating the adaptive responses to stressful events at early exposure in the uterine environment [39]. In this perspective, nevertheless, the exact role of the maternal environment during pregnancy in regulating the placental epigenome, thus potentially affecting fetal growth and establishing the basis for the inevitable effects in postnatal life, remains to be clarified. 

The main weakness of this study is the placental sample size, which may have limited our power to detect associations, although it should be noted that we assessed the maternal DNA methylation profiles and gene expression in placentas as well as DNA methylation in blood. Many DNA methylation patterns are tissue-specific and cell-specific; thus, differences in methylation across tissues are critical and are key to understanding the role of epigenetics in NCDs [40].

In this view, the strength of this study is that we assessed DNA methylation levels on some tissue samples, including blood, buccal swab samples, and placenta, and it is more informative.

In fact, peripheral blood and buccal swab samples are a more practical DNA source because they are easily accessible, and their collection is acceptable to patients [41]. In particular, buccal DNA samples can be collected through non-invasive protocols, and their collection is more readily at birth, relatively inexpensive, safe, and easy [42]. In conclusion, pregnancy, which is a period of physical, hormonal, and humoral changes, represents a unique time period to define the future health of both mother and offspring [43]. In line with other previous studies [44], our findings suggest that maternal hyperglycemia and obesity can induce effects on the newborn via epigenetic variations, providing new insights into fetal metabolic programming.

When the alterations of DNA methylation occur in utero or during the early neonatal stages, these epigenetic signatures can act as causative agents for effects on health. 

Therefore, we suggest that adverse intrauterine environments, such as hyperglycemia and maternal obesity during pregnancy, can affect the DNA methylation pattern of promoters of *LPL,* involved in glucose and lipid metabolism, as well as *MC4R*, involved in obesity and weight gain, both in mothers and their children, thereby providing a potential mechanism for long-lasting effects. Epigenetic modifications in *MC4R* and *LPL* will need to be assessed in these offspring over time in order to also evaluate the possible consequences throughout infancy and childhood. In this view, our findings showed that epigenetics offers valuable insights into metabolic programming and can provide novel biomarkers for early disease prediction. Furthermore, understanding the epigenetic mechanisms in conjunction with lifestyle modifications can guide future research directions in clinical practice, including the development of effective prevention measures beyond disease prediction information.

## 7. Conclusions

In summary, our results suggest that *MC4R* and *LPL* methylation patterns can influence fetal programming in response to maternal metabolic status in pregnancy. Thus, in the future, it is necessary to establish the long-lasting effects in the offspring triggered by these epigenetic perturbations.

## Figures and Tables

**Table 1:** Assay design for pyrosequencing of the DNA methylation level at the *MC4R* and *LPL* gene promoter loci.

Gene	Primers	Product Size (pb)	Number of CpGs Analyzed
*MC4R*	F: 5′-AGGGTGATATAGATTTAGATGTAGAAGT-3′ R:5′-[Btn]AAACAATATACTTTCCATTTCATTTTACAC-3′ Seq: 5′-GTAGAAGTTTTTGAAGTTTG-3′	202	2
*LPL*	F: 5′-AAGTATAAGTTGGGAAGTAATGTGTG-3′ R: 5′bio-CCAAAAAAAAAAAATTTAACTATTAAATTAC-3′ Seq: 5′-GTGTGTTTTTTTATTTTTATATTGA-3′	177	4

**Table 2:** Clinical characteristics of NGT vs. GDM and NW vs. OB women.

Characteristics	NGT (n = 17)	GDM (n = 84)	*p*-Value	NW (n = 55)	OB (n = 46)	*p*-Value
Age (years)	33.7 (5.3)	35.3 (4.9)	0.221 ^+^	35.6 (4.3)	34.4 (5.6)	0.208 ^+^
Systolic blood pressure (mmHg)	112.2 (11.58)	117.5 (14.4)	0.186 ^+^	112.2 (11.6)	121.2 (15.1)	**0.005** ^+^
Diastolic blood pressure (mmHg)	68.1 (7.0)	70.8 (9.0)	0.273 ^+^	67.7 (7.9)	73.3 (8.6)	**0.005** ^+^
PREDIMED	8.0 (7–9)	9.0 (8–10)	**0.037** ^$^	9.5 (9–10)	9.0 (8–10)	**0.004** ^$^
PREDIMED CLASS *			0.061 ^§^			**0.013** ^§^
No adherence	2 (13.3%)	2 (2.6%)		0 (0.0%)	4 (9.3%)	
Medium adherence	10 (66.7%)	42 (53.8%)		25 (50.0%)	27 (62.8%)	
Maximum adherence	3 (20.0%)	34 (43.6%)		25 (50.0%)	12 (27.9%)	
IPAQ *			0.824 ^§^			0.427 ^§^
Low	3 (20.0%)	21 (26.9%)		13 (26.0%)	11 (25.6%)	
Moderate	5 (33.3%)	28 (35.9%)		15 (30.0%)	18 (41.9%)	
High	7 (46.7%)	29 (37.2%)		22 (44.0%)	14 (32.6%)	
Smoking history *			0.339 ^§^			0.118 ^§^
Non-smoker	5 (41.7%)	48 (60.0%)		33 (66.0%)	20 (47.6%)	
Smoker	0 (0.0%)	3 (3.8%)		2 (4.0%)	1 (2.4%)	
Ex-smoker	7 (58.3%)	29 (36.2%)		15 (30.0%)	21 (50.0%)	
Pre-pregnancy weight (Kg)	80.3 (23.8)	70.9 (19.5)	0.083 ^+^	58.2 (6.3)	89.5 (18.3)	**<0.001** ^+^
Weight at the end of pregnancy (Kg)	92.5 (22.0)	80.5 (17.8)	**0.019** ^+^	70.0 (7.7)	97.0 (17.8)	**<0.001** ^+^
Pre-pregnancy BMI (Kg/m^2^)	29.2 (7.8)	26.6 (7.6)	0.202 ^+^	21.8 (1.9)	33.3 (7.3)	**<0.001** ^+^
BMI at the end of pregnancy (Kg/m^2^)	33.6 (7.2)	30.2 (6.9)	0.072 ^+^	26.2 (2.4)	36.1 (6.9)	**<0.001** ^+^
Weight variation (Kg)	10.7 (5.8)	9.6 (6.7)	0.562 ^+^	11.7 (5.9)	7.5 (6.6)	**<0.001** ^§^
Delivery *			0.964 ^§^			0.163 ^§^
Vaginal delivery	10 (62.5%)	53 (63.1%)		38 (69.1%)	17 (30.9%)	
Cesarean section	6 (37.5%)	31 (36.9%)		25 (55.6%)	20 (44.4%)	
Third-trimester TC (mg/dL)	225.6 (30.3)	254.3 (46.3)	**0.049** ^+^	249.9 (45.9)	251.6 (45.4)	0.865 ^+^
Third-trimester HDL-C (mg/dL)	60.6 (11.5)	64.7 (13.2)	0.336 ^+^	68.1 (12.7)	59.2 (11.7)	**0.001** ^+^
Third-trimester TG (mg/dL)	187.8 (86.1)	230.8 (126.1)	0.278 ^+^	207.5 (103.6)	247.9 (140.4)	0.128 ^+^
Third-trimester LDL-C (mg/dL)	127.4 (22.0)	145.8 (35.3)	0.097 ^+^	140.3 (35.3)	147.5 (33.0)	0.344 ^+^
OGTT (mg/dL) at baseline (min)	83.0 (4.4)	90.8 (9.3)	**<0.001** ^+^	86.6 (9.2)	93.0 (7.8)	**<0.001** ^+^
OGTT (mg/dL) after 60 min	131.5 (25.7)	162.4 (34.0)	**<0.001** ^+^	155.6 (37.3)	158.8 (31.4)	0.646 ^+^
OGTT (mg/dL) after 120 min	115.9 (20.0)	135.7 (33.2)	**0.019**	132.7 (34.1)	131.9 (30.1)	0.908
First quarter fasting blood glucose (mg/dL)	86.6 (11.6)	85.9 (8.9)	0.801 ^+^	86.0 (8.6)	85.9 (10.1)	0.980 ^+^

Statistically significant values are in bold. Data are expressed as mean and standard deviation (SD) or median and interquartile range (IQR), absolute frequency (n), and column percentage (%). ^§^ *p*-value derived from Chi-squared test or Fisher exact test; ^+^ *p*-value derived from unpaired Student *t*-test or ^$^ Wilcoxon rank test. * The data refer to the available information. TC: total cholesterol; HDL-C: high-density lipoprotein cholesterol; LDL-C: low-density lipoprotein cholesterol; TG: triglycerides; OGTT: oral glucose tolerance test.

**Table 3:** Neonatal outcomes relative to comparison between NGT vs. GDM and NW vs. OB women.

Characteristics	NGT (n = 17)	GDM (n = 84)	*p*-Value	NW (n = 55)	OB (n = 46)	*p*-Value
Gestational week	39.1 (1.4)	38.5 (1.2)	**0.047** ^+^	38.9 (1.3)	38.2 (1.0)	**0.014** ^+^
Gender *			0.300 ^§^			0.503 ^§^
Male	11 (68.8%)	46 (54.8%)		33 (60.0%)	24 (53.3%)	
Female	5 (31.2%)	38 (45.2%)		22 (40.0%)	21 (46.7%)	
Birth weight (grams)	3348.8 (289.5)	3190.4 (454.7)	0.183 ^+^	3191.8 (453.5)	3244.9 (415.0)	0.546 ^+^
One-minute Apgar scores	7.9 (1.7)	8.8 (0.7)	**<0.001** ^+^	8.8 (0.5)	8.4 (1.3)	**0.028** ^+^
Five-minute Apgar scores	9.2 (0.9)	9.73 (0.7)	**<0.001** ^+^	9.8 (0.5)	9.6 (0.7)	0.061 ^+^
Birth head circumference (cm)	34.5 (1.6)	34.7(1.8)	0.689 ^+^	34.6 (1.8)	34.0 (1.7)	0.124 ^+^
Birth length (cm)	50.2 (1.3)	49.6 (2.0)	0.222 ^+^	49.9 (2.0)	49.5 (1.7)	0.374 ^+^
Hypoglycemia *			**0.050** ^§^			0.993 ^§^
No	13 (81.2%)	43 (55.1%)		31 (59.6%)	25 (59.5%)	
Yes	3 (18.8%)	35 (44.9%)		21 (40.4%)	17 (40.5%)	

Statistically significant values are in bold. Data are expressed as mean and standard deviation (SD), absolute frequency (n), and column percentage (%). ^§^ *p*-value derived from Chi-squared test or Fisher exact test; ^+^ *p*-value derived from unpaired Student *t*-test ^§^. * The data refer to the available information.

**Table 4:** *MC4R* and *LPL* DNA methylation levels in newborns and women.

DNA Methylation%	NGT (n = 17)	GDM (n = 84)	*p*-Value	NW (n = 55)	OB (n = 46)	*p*-Value
**MC4R Women**						
CpG1	9.4 (6.3)	8.4 (5.8)	0.541	8.3 (4.7)	8.8 (7.1)	0.674
CpG2	15.2 (9.46)	13.5 (4.6)	0.254	13.6 (4.6)	13.9 (6.9)	0.839
Mean methylation levels	12.1 (7.9)	10.9 (4.7)	0.426	11.0 (4.5)	11.3 (6.3)	0.778
**Newborns**						
CpG1	6.9 (6.2)	2.8 (3.0)	**<0.001**	3.4 (3.4)	3.7 (4.5)	0.672
CpG2	6.8 (5.6)	3.8 (3.3)	**0.003**	4.2 (3.8)	4.3 (4.1)	0.917
Mean methylation levels	6.8 (5.8)	3.4 (3.0)	**<0.001**	3.8 (3.4)	4.1 (4.4)	0.690
**LPL Women**						
CpG1	23.8 (10.7)	23.2 (6.9)	0.768	23.7 (6.9)	22.8 (8.4)	0.570
CpG2	15.7 (7.7)	14.0 (6.6)	0.381	14.3 (6.8)	14.3 (6.8)	0.957
CpG3	20.8 (9.0)	25.1 (12.3)	0.182	24.4 (13.2)	24.4 (10.3)	0.990
CpG4	45.9 (15.6)	43.5 (14.1)	0.543	42.6 (13.9)	45.5 (14.8)	0.328
Mean methylation levels	26.5 (7.0)	26.5 (7.6)	0.985	26.2 (7.9)	26.8 (7.0)	0.728
**Newborns**						
CpG1	12.6 (5.0)	13.8 (5.4)	0.431	13.5 (5.4)	13.7 (5.4)	0.877
CpG2	9.1 (4.7)	10.6 (8.0)	0.472	10.7 (7.3)	9.8 (7.9)	0.559
CpG3	12.6 (8.5)	13.9 (10.2)	0.613	13.5 (9.5)	13.9 (10.5)	0.839
CpG4	26.2 (17.0)	25.0 (12.0)	0.718	24.6 (11.2)	25.8 (14.8)	0.637
Mean methylation levels	15.1 (5.9)	15.8 (7.5)	0.704	15.6 (6.8)	15.8 (7.7)	0.934

Statistically significant values are in bold. Data are expressed as mean and standard deviation (SD). *p*-values are derived from unpaired Student *t*-tests.

## Data Availability

All data generated or analyzed during this study are included in this published article.

## Supplementary Materials

The following supporting information can be downloaded at https://www.mdpi.com/article/10.3390/nu16203502/s1. Table S1: *MC4R* and *LPL* DNA methylation levels on the maternal and fetal side of placenta in NGT vs GDM and NW vs OB women.
