# Supplementary material for: From Mother to Child: Epigenetic Signatures of Hyperglycemia and Obesity during Pregnancy
Source: Nutrients. 2024 Oct 16;16(20):3502. doi: 10.3390/nu16203502 (PMC11510513; doi:10.3390/nu16203502)
Supplement: Supplementary file 1 [file nutrients-16-03502-s001.zip › nutrients-3237524-supplementary.pdf]

Supplementary Table S1. *MC4R* and *LPL* DNA methylation levels on the maternal and fetal side of placenta in NGT vs. GDM and NW vs. OB women.

**DNA Methylation %**	**NGT (n=19)**	**GDM (n=21)**	***p-value***	**NW (n=23)**	**OB (n=17)**	***p-value***
MC4R						
Maternal side of placenta						
CpG1	12.9 (7.3)	10.3 (7.3)	0.264	11.9 (8.9)	11.1 (4.7)	0.742
CpG2	19.1 (9.9)	18.3 (9.9)	0.794	17.3 (9.8)	20.5 (9.8)	0.305
Maternal mean methylation levels	16.4 (7.6)	14.2 (8.2)	0.394	14.8 (8.8)	15.8 (6.7)	0.685
Fetal side of placenta						
CpG1	14.1 (9.2)	12.5 (8.3)	0.563	15.0(10.0)	11.0 (6.0)	0.147
CpG2	22.6 (12.1)	21.0 (8.1)	0.622	21.5 (8.8)	22.1(11.9)	0.860
Fetal mean methylation levels	18.3 (10.0)	16.7 (7.8)	0.579	18.2 (9.1)	16.5 (8.7)	0.557
LPL						
Maternal side of placenta						
CpG1	28.9 (11.5)	27.4 (11.7)	0.679	27.7 (10.5)	28.6 (13.0)	0.815
CpG2	19.0 (10.8)	14.6 (6.9)	0.135	17.8 (9.8)	15.1 (8.0)	0.357
CpG3	30.1(13.1)	31.4 (12.2)	0.738	30.3(13.9)	31.5 (10.8)	0.759
CpG4	53.1 (11.8)	53.2 (18.8)	0.987	52.3(18.9)	54.4 (10.2)	0.679
Mean methylation levels	32.4 (7.2)	33.8 (11.7)	0.666	32.6 (8.5)	33.9 (11.3)	0.696
Fetal side of placenta						
CpG1	28.1 (9.9)	33.2 (14.7)	0.213	30.7 (12.9)	30.8 (13.0)	0.974
CpG2	18.4 (9.5)	19.9 (10.6)	0.634	17.9 (9.2)	21.0 (11.0)	0.347
CpG3	38.9 (11.3)	38.5 (19.7)	0.934	37.8 (14.0)	39.9 (18.9)	0.683
CpG4	60.8 (16.3)	59.4 (15.2)	0.786	55.7 (15.2)	66.0 (14.4)	**0.037**

Mean methylation levels	36.1 (8.2)	37.8 (9.8)	0.570	35.5 (7.1)	38.9 (11.0)	0.241

Statistically significant values are in bold.. Data are expressed as mean and standard deviation (SD).

+p-value derived from unpaired Student T-test.
